# *Usp14* down-regulation corrects sleep and circadian dysfunction of a Drosophila model of Parkinson’s disease

**DOI:** 10.3389/fnins.2024.1410139

**Published:** 2024-08-05

**Authors:** Mariavittoria Favaro, Sofia Mauri, Greta Bernardo, Mauro A. Zordan, Gabriella M. Mazzotta, Elena Ziviani

**Affiliations:** Department of Biology, University of Padova, Padova, Italy

**Keywords:** USP14, PINK1, Drosophila, circadian clock, sleep, mitochondrial fission

## Abstract

PD is a complex, multifactorial neurodegenerative disease, which occurs sporadically in aged population, with some genetically linked cases. Patients develop a very obvious locomotor phenotype, with symptoms such as bradykinesia, resting tremor, muscular rigidity, and postural instability. At the cellular level, PD pathology is characterized by the presence of intracytoplasmic neurotoxic aggregates of misfolded proteins and dysfunctional organelles, resulting from failure in mechanisms of proteostasis. Nonmotor symptoms, such as constipation and olfactory deficits, are also very common in PD. They include alteration in the circadian clock, and defects in the sleep–wake cycle, which is controlled by the clock. These non-motor symptoms precede the onset of the motor symptoms by many years, offering a window of therapeutic intervention that could delay—or even prevent—the progression of the disease. The mechanistic link between aberrant circadian rhythms and neurodegeneration in PD is not fully understood, although proposed underlying mechanisms include alterations in protein homeostasis (proteostasis), which can impact protein levels of core components of the clock. Loss of proteostasis depends on the progressive pathological decline in the proteolytic activity of two major degradative systems, the ubiquitin-proteasome and the lysosome-autophagy systems, which is exacerbated in age-dependent neurodegenerative conditions like PD. Accordingly, it is known that promoting proteasome or autophagy activity increases lifespan, and rescues the pathological phenotype of animal models of neurodegeneration, presumably by enhancing the degradation of misfolded proteins and dysfunctional organelles, which are known to accumulate in these models, and to induce intracellular damage. We can enhance proteostasis by pharmacologically inhibiting or down-regulating Usp14, a proteasome-associated deubiquitinating enzyme (DUB). In a previous work, we showed that inhibition of Usp14 enhances the activity of the ubiquitin-proteasome system (UPS), autophagy and mitophagy, and abolishes motor symptoms of two well-established fly models of PD that accumulate dysfunctional mitochondria. In this work we extended the evidence on the protective effect of Usp14 down-regulation, and investigated the beneficial effect of down-regulating Usp14 in a Pink1 Drosophila model of PD that develop circadian and sleep dysfunction. We show that down-regulation of Usp14 ameliorates sleep disturbances and circadian defects that are associated to Pink1 KO flies.

## 1 Introduction

Parkinson’s disease (PD) is the second most common degenerative disease of the central nervous system, which affects around 0.3% of the population (Raza et al., 2019). The pathological hallmarks include accumulation of filamentous, cytoplasmic inclusions consisting mainly of α-synuclein aggregations in the form of Lewy bodies (LB) in the *substantia nigra* of the brain, and the selective loss of dopaminergic neurons with consequently depleted dopamine levels. These deficiencies lead to motor dysfunction, with symptoms such as bradykinesia, resting tremor, muscular rigidity, and postural instability (Kalia and Lang, 2015).

PD is mainly a sporadic disorder of largely unknown etiology, which in most cases affects the elderly. The disease can result from genetic and environmental factors, although approximately 10% of all cases have a clear genetic origin, and show early manifestation, with currently at least 23 disease-segregating loci identified (Hernandez et al., 2016). Genetic and sporadic forms manifest similar clinical manifestation and indistinguishable PD hallmarks. Therefore, sporadic PD will likely benefit from studies of the molecular pathways which are impaired in familiar cases.

The identification and the study of these rare genetic PD forms identified oxidative stress, mitochondrial dysfunction, and alteration of the ubiquitin-proteasome system (UPS) and autophagy as causal factors in the pathophysiology of PD (Kalia and Lang, 2015). Most of the proteins encoded by PD genes (PINK1, Parkin and DJ1 in particular) are important for mitochondrial homeostasis and function. Accumulation of dysfunctional mitochondria can indeed contribute to cell demise in different ways. Defective mitochondria can enhance oxidative stress (Blesa et al., 2015), which exacerbates protein misfolding. They can also lead to inflammation derived from mitochondrial release of damage associated molecular patterns (mtDAMPs; Sliter et al., 2018), and impair calcium (Ca^2+^) homeostasis (Giorgi et al., 2018). PINK1, Parkin and DJ1 play a fundamental role in preventing damage originating from dysfunctional mitochondria. In particular, PINK1 and Parkin operate within the same pathway to induce the degradation of damaged mitochondria via autophagy (Narendra et al., 2010) and mitigate inflammation by repressing mitochondrial antigen presentation (Matheoud et al., 2016), while DJ1 exerts neuroprotective effects by enhancing the antioxidant defense of the cell (Kalia and Lang, 2015).

In addition to the above-mentioned primary motor function impairments, non-motor features are also frequently present in PD. Sleep disorders in particular are found in two-thirds of PD patients, and they include symptoms like insomnia, vivid dreaming, increased nocturnal activity, more fragmented sleep, REM sleep behavior disorder (RBD) and excessive daytime sleepiness (Raza et al., 2019). Because sleep behavior is controlled by circadian rhythmicity, sleep disorders can be a consequence of dysfunctional circadian clock in PD patients. Indeed, although circadian disorders are not frequently mentioned among PD symptoms, several studies in rodent PD models have shown a correlation between PD-associated neurodegenerative phenotype and circadian dysfunction (Simola et al., 2007; Hunt et al., 2022; Summa et al., 2024).

Sleep–wake cycle is controlled by the circadian clock, an internal timing system that allows adapting to and anticipating the daily changes that occur on planet Earth. It is composed by three major elements: a core oscillator, which generates the rhythmicity; inputs pathways that allow the organism to perceive the external stimuli and synchronize the core oscillator; and output pathways (represented by the circadian phenotypes), generated by the cyclic expression of clock-controlled output genes (Patke et al., 2020). At the cellular level, the circadian clock is identified by a specific subset of highly specialized neurons, called central pacemaker, which in mammals are localized in the suprachiasmatic nucleus (SCN) of the anterior hypothalamus. These neurons receive inputs from daylight, which is used to synchronize the approx. 24 h rhythm of the internal clock to the exact 24 h daily rhythm of planet Earth, to optimize the performance of the entire organism (Patke et al., 2020). At the molecular level, circadian oscillations are generated by evolutionary conserved interlocked transcriptional-translational feedback loops (TTFLs), which take approximately 24 h to complete. In TTFLs, positive elements promote the rhythmic transcription of the negative elements that inhibit in a feedback loop the activity of the positive elements. This is a self-sustained, cell autonomous mechanism generating the circadian rhythm in the SCN, which in fact persists even in the absence of light (Patke et al., 2020). In Drosophila, the circadian oscillation is based on two main interlocked TTFLs: in the primary loop, the transcription factors Clock (Clk) and cycle (cyc) control the rhythmic expression of the negative elements period (per) and timeless (tim), while the second loop results in the rhythmic expression of *Clk* (Hardin, 2011).

As expected, disruption of the rhythmicity can lead to the development of diseases, such as metabolic syndromes, cancer and cardiovascular diseases, and contribute to the onset of neurodegenerative diseases (Xie et al., 2019). Thus, the maintenance of proper network coordination within the clock is essential for health and well-being.

The molecular mechanism underlying the link between disturbed circadian rhythms and neurodegeneration is not known, but alteration in protein homeostasis (proteostasis) seems to be a plausible connection (Leng et al., 2019). In that respect, many studies highlighted the relevance of clock-dependent regulation of general proteostasis (Kang et al., 2009) and autophagy (Ma et al., 2011; Ryzhikov et al., 2019), and the physiological importance of sleeping as a mean to degrade proteotoxic compounds and insoluble aggregates that accumulate during the active phase (Varshavsky, 2012; Xie et al., 2013; Shokri-Kojori et al., 2018), and may induce intracellular damage.

In this study we want to exploit one well-established Drosophila model of PD that manifest abnormal circadian rhythmicity and disturbed sleep–wake cycle, to investigate the effect of enhancing proteostasis in the context of circadian and sleep disturbances. We can enhance proteostasis by down-regulating deubiquitinating enzyme (DUB) Usp14. Usp14 is a DUB, belonging to ubiquitin-specific processing (USP) family, which negatively regulate the ubiquitin-proteasome system (UPS), autophagy and mitophagy. Hence, its inhibition is predicted to both enhance the activity of the UPS, autophagy and mitophagy. Previous studies have shown that genetic down-regulation of *Usp14* or its pharmacological inhibition by specific inhibitor IU1, recovered locomotor behavior of *Pink1* KO flies, and it extended flies’ lifespan, presumably by enhancing protein and organelle homeostasis (Chakraborty et al., 2018). With that in mind, this work wants to test the effect of down-regulating *Usp14* in *Pink1* KO flies in the context of circadian rhythmicity and sleep–wake cycle.

Our results show that down-regulation of *Usp14* ameliorates sleep disturbances and circadian defects that are associated to *Pink1* KO flies.

## 2 Materials and methods

### 2.1 Fly strains and husbandry

Flies were raised under standard conditions at 23°C with a 12:12 h light:dark cycle (unless differently stated), on agar, cornmeal, yeast food. Wild type (*w^1118^*) and driver lines (*GAL4*) were obtained from Bloomington Drosophila Stock Center. *Pink1^B9^* mutants have been described before (Park et al., 2006; Supplementary Table 1), and were provided by Dr. A. Whitworth. The *UAS-Usp14 RNAi* line (KK-110227) was obtained from VDRC Stock Center.

### 2.2 RNA extraction, reverse transcription and qPCR

For RNA extraction, whole flies were homogenized in TRI Reagent (Zymo Research), and RNA was extracted following manufacturer’s instruction. For each sample, 1 μg of RNA was used for first-strand cDNA synthesis with the SensiFAST cDNA Synthesis Kit (Bioline) following manufacturer instruction. Three technical replicates were loaded for each sample and the expression level of *RpL32* was set as an internal amplification control for normalization. qRT-PCRs were performed using the HOT FIREPol SolisGreen qPCR Mix (Solis Biodyne) on a QuantStudio 5 Real-Time PCR System (Thermofisher; Usp14_FW AGCTCAGAAGAGGATCCCGA and Usp14_RV CGGCTCACCAAGTAAGTTCG; RpL32_FW: ATCGGTTACGGATCGAACAA and RpL32_RV: GACAATCTCCTTGCGCTTCT).

### 2.3 Assessment of locomotor activity: Drosophila activity monitoring system

#### 2.3.1 Circadian analysis

Locomotor activity of 3–10 days old individual adult male flies was recorded for 3 days in light–dark conditions (LD) and 7 days in constant dark conditions (DD) using the Drosophila Activity Monitoring System (TriKinetics, Inc., Waltham, MA, United States). Data were collected every 1 min and then analyzed in 30 min bins using the FaasX (Fly Activity Analysis Suite for MacOS) software, developed by M. Boudinot and François Rouyer (Institut de Neurobiologie Alfred Fessard, CNRS, France). Rhythmic flies were defined by chi-square periodogram analysis with a power of ≥20. Values for individual flies were pooled to obtain an average value for each independent line analyzed. Morning Index was calculated for each fly using the following mathematical approach (Seluzicki et al., 2014):

$M.Index=\frac{\left(\sum activity.3h.before.light.on\right)}{\left(\sum activity.6h.before.light.on\right)}-0.5$

#### 2.3.2 Sleep analysis

The activity data collected by the DAMS (Trikinetics, United States) activity monitors were analyzed using custom R scripts (Mauro Zordan_Department of Biology, University of Padova). The scripts were designed to access the data files generated by the DAMS activity software and prepare the data for inspection, for the generation of graphs (based on the ggplot2 package by Hadley Wickham) and for statistical analysis (based on ANOVA with Tukey HSD correction for multiple comparisons).

### 2.4 Statistical analysis

Statistical analysis was performed using GraphPad PRISM as described in detail below.

### 2.4.1 Analysis of activity

Statistical analysis of circadian activity was performed using GraphPad PRISM. Statistical significance was determined by two-tailed Student’s *t-*test. Each bar of the bar charts (total activity and period) represents mean ± SEM of the indicated parameter for the indicated genotype. Bar charts underlying binomial distributions (rhythmicity, EA and MA) represent the percentage of flies showing the indicated phenotype for each genotype. Standard Deviation of a binomial distribution was calculated with the following formula: σ=SQRT(n*p*q) where: *n* = number of flies; *p* = The probability of success (expressed in decimal); *q* = the probability of failure (expressed in decimal). Significance was determined by Contingency Fisher’s exact test. *p < 0.05; **p < 0.01; ***p < 0.001; ****p < 0.0001. “n” indicates the number of flies analyzed per genotype.

### 2.4.2 Analysis of sleep

Statistical analysis of sleep behavior was performed using GraphPad PRISM. Significance was determined by two-tailed Student’s *t-*test or Tukey’s HSD multiple comparison test. For each box plot, the median is shown with the upper and lower quartile and the 0^th^ and 100^th^ percentile; *p < 0.05; **p < 0.01; ***p < 0.001.

## 3 Results

### 3.1 Down-regulation of *Usp14* slightly lengthens the circadian period of *Drosophila melanogaster*

Usp14 is a DUB intrinsically associated with the proteasome that belongs to the USP family. Usp14 suppresses the degradation of its targets by promoting the dissociation of the substrate from the proteasome before the commitment step. Hence, inhibition of Usp14 stimulates proteasome-dependent degradation of its substrates, and it can overall enhance protein degradation (Lee et al., 2010). In mammals, Usp14 appears to be also a clock-relevant DUB that contributes to the maintenance of the circadian clock. In particular, Usp14 activity affects the circadian period by slowing down the degradation of clock protein PER. Accumulation of PER generates a time delay in the removal of the inhibition of CLOCK and BMAL1 (the mammalian counterpart of the Drosophila cyc), which is required for the maintenance of a robust circadian clock (D'Alessandro et al., 2017).

**Figure 1:**
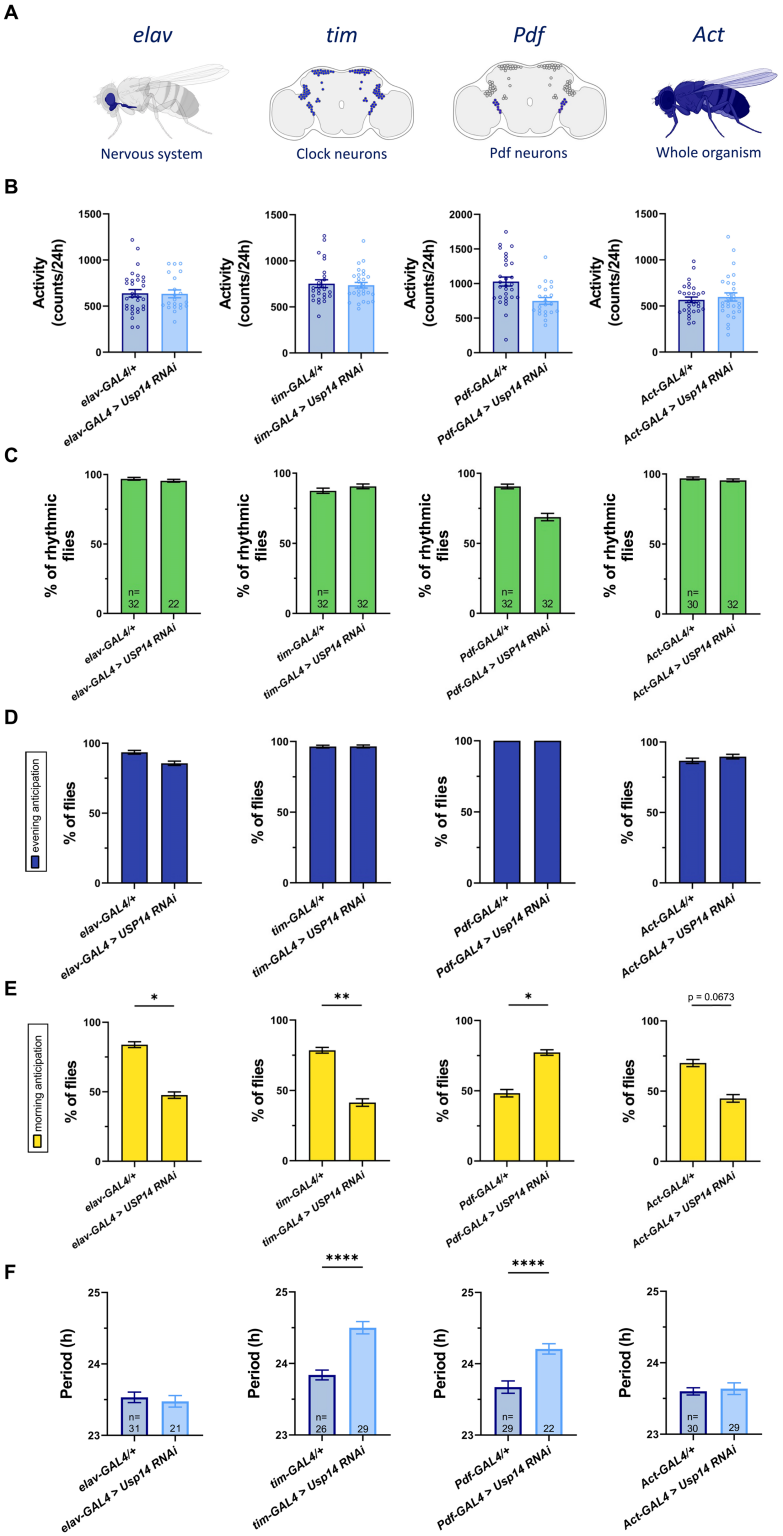
Cell-specific genetic down-regulation of *Usp14* lengthens the circadian period of *Drosophila melanogaster*, and partially affects their Morning Anticipation. **(A)** Diagram showing neurons and tissues targeted by Usp14 RNAi using elav-GAL4, tim-GAL4, Pdf-GAL4 and Act-GAL4, respectively. **(B)** Bar charts showing total activity during the second day of locomotor activity recording in LD 12:12 conditions (12h of light and 12h of darkness). Bar graph expresses mean ± SEM of total activity of the indicated genotypes measured as the number of time that the fly intersected the infrared beam of the recording apparatus in 24hrs. **(C)** Bar charts showing percentage of rhythmic flies. **(D)** Bar charts indicating the percentage of flies that show evening anticipation (EA). EA was calculated as described in materials and methods. **(E)** Bar charts indicating the percentage of flies that show morning anticipation (MA). MA was calculated as described in materials and methods. **(F)** Bar charts showing the length of the period of rhythmic flies for the indicated genotype. Analysis was performed on rhythmic flies for **(B)**, **(D)** and **(E)**, and number of flies for each genotype is indicated in **(F)**.

That considered, we first wanted to address whether Usp14 could similarly affect the circadian clock in flies. To test this, we developed and validated a *UAS/GAL4*-based RNAi approach (Hales et al., 2015) for cell-specific down-regulation of *Usp14* in specific tissues (Supplementary Figure 1). We used four different drivers (*elav*, *tim*, *Pdf* and *Actin*) to express *Usp14 RNAi* (i.e., down-regulate *Usp14*) in different target cells (Figure 1A). In all conditions, we did not record any developmental defect when *Usp14* was down-regulated. Locomotor activity and circadian behavior of the abovementioned transgenic flies was recorded using the TriKinetics Drosophila Activity Monitor (DAM; Rosato and Kyriacou, 2006; Schlichting et al., 2016; Julienne et al., 2017).

We found that down-regulation of *Usp14* in the tested tissues did not affect total activity of the flies (Figure 1B), nor rhythmic behavior in LD conditions (Figure 1C). A similar lack of effect was observed for evening anticipation (EA; i.e. increased activity just before the transition to lights-off; Figure 1D), whereas morning anticipation (MA; i.e. increased activity just before the transition to lights-on) was only slightly affected in flies in which *Usp14* was selectively down-regulated (Figure 1E). While *Usp14* KD did not seem to significantly affect the overall circadian behavior of flies in terms of total activity, rhythmicity, and light switch anticipation, specific knockdown of *Usp14* in tim + and Pdf + cells slightly lengthened the period of flies from 23.8 h to 24.5 h and from 23.7 h to 24.2 h, respectively (Figure 1F).

In summary, down-regulation of *Usp14* in tim + and Pdf + cells slightly lengthened the circadian period of flies. This effect was specific for tim and Pdf expressing neurons, as it was not observed in other neuronal cells. The other circadian parameters that we tested (rhythmicity, total activity and light switch anticipation) did not seem to be largely affected by *Usp14* down-regulation.

### 3.2 Drosophila *Pink1* mutant (KO) flies display reduced total activity, weakened circadian rhythms, and altered sleep behavior

**Figure 2:**
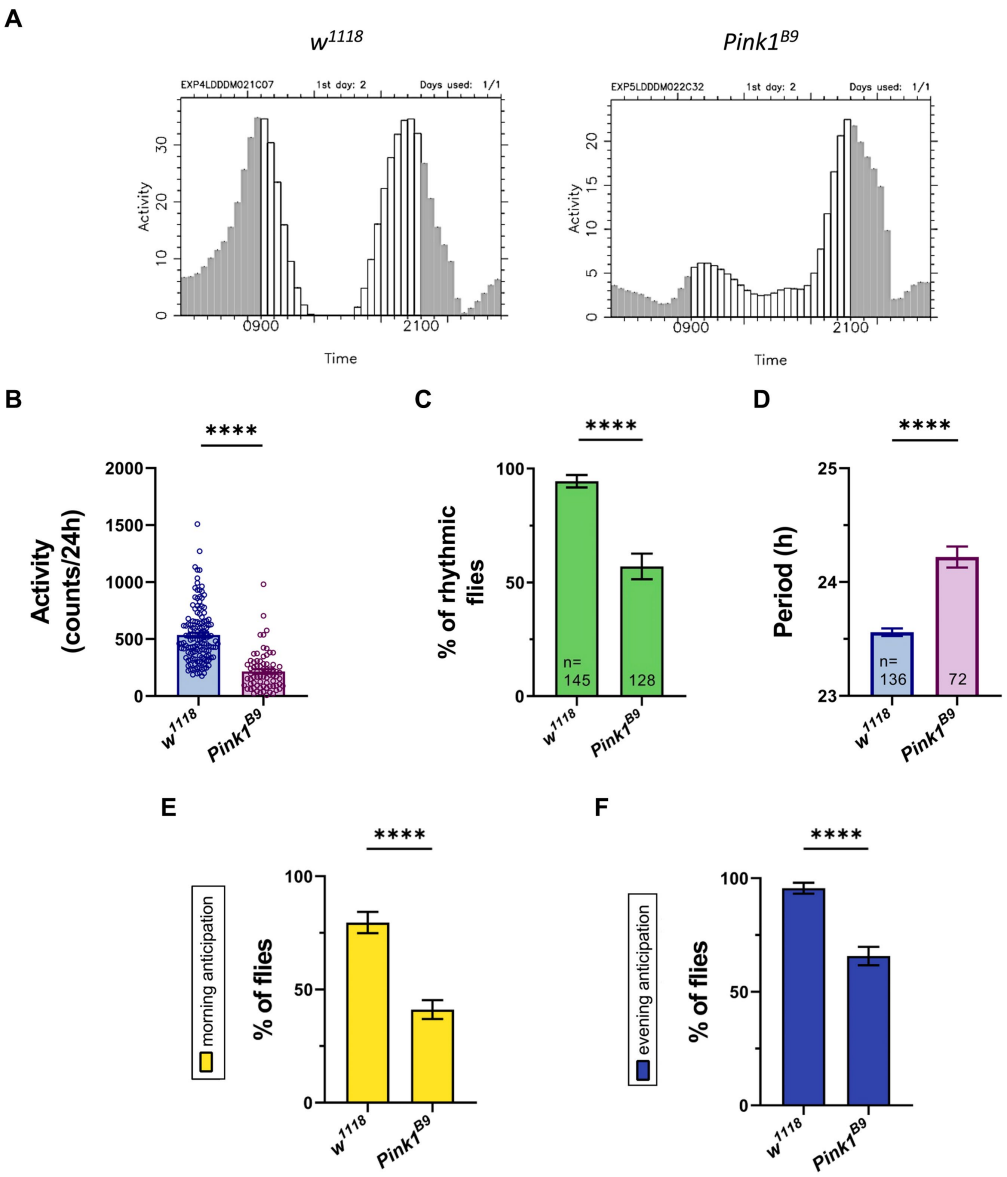
Drosophila *Pink1* mutant (KO) flies display aberrant circadian rhythmicity, with lower levels of activity during the day, and disrupted morning and evening anticipation. **(A)** Representative education charts showing the activity of a single fly in the second day of LD 12:12 conditions (12 h of light and 12 h of darkness). Each bar represents the activity counts every 30 minutes. **(B)** Bar charts showing total activity during the second day of locomotor activity recording in LD. Charts show total activity for each genotype as beam crosses per 24hrs. **(C)** Bar charts showing percentage of rhythmic flies. Number of total flies (rhythmic and arrhythmic) for each genotype is indicated. **(D)** Bar charts showing the period of rhythmic mutant flies. **(E)** Bar charts indicating the percentage of flies that show morning anticipation (MA). **(F)** Bar charts indicating the percentage of flies that show evening anticipation (EA). Analysis was performed on rhythmic flies for **(B)**, **(E)** and **(F)**, and number of flies for each genotype is indicated in **(D)**.

Published works indicate that *Pink1* KO flies manifest abnormal circadian rhythmicity and disrupted sleep–wake cycle, with some discrepancies between studies (Julienne et al., 2017; Doktor et al., 2019). Thus, we wanted to explore in depth circadian rhythmicity and total activity of these flies, to strengthen Drosophila as animal model for investigating non-motor symptoms of PD. To this aim, we used *Pink1^B9^* null strains, which were extensively used before to study PD motor phenotype. In particular, *Pink1^B9^* null mutant flies display phenotypes such as abnormal wing posture, degeneration of flight muscles, mitochondrial dysfunction and loss of dopaminergic neurons, which are highly reproducible, and translate into climbing and flight defects (Greene et al., 2003; Park et al., 2006). We monitored locomotor activity of wild type and *Pink1^B9^* mutant flies by using the DAM system. Flies were kept in light/dark (LD) conditions for 3 days, and then shifted to constant darkness (DD) for 7 days to monitor endogenous circadian locomotor activity. Wild type flies displayed morning and evening peaks in activity, with characteristic anticipation before lights-on (morning anticipation, MA) or lights-off (evening anticipation, EA). They were active during the day and relatively inactive at night (Figure 2A). They displayed a total activity of about 500 beam-recorded counts per day (Figure 2B), and maintained circadian rhythmicity (Figure 2C), with a period of 23.6 h (Figure 2D). Most of them displayed morning and evening anticipation (Figures 2E,F). On the other hand, *Pink1^B9^* null mutants displayed reduced total activity (Figure 2B). In constant conditions, a higher percentage of *Pink1^B9^* flies became arrhythmic (Figure 2C). Among those flies that remained rhythmic, we recorded a significant lengthening of the circadian period compared to wild type flies (from 23.6 h to 26.2 h; Figure 2D). Also, *Pink1^B9^* flies show impairment in their ability to anticipate light transitions, and a significantly smaller percentage of them displayed morning (Figure 2E) and evening anticipation (Figure 2F).

**Figure 3:**
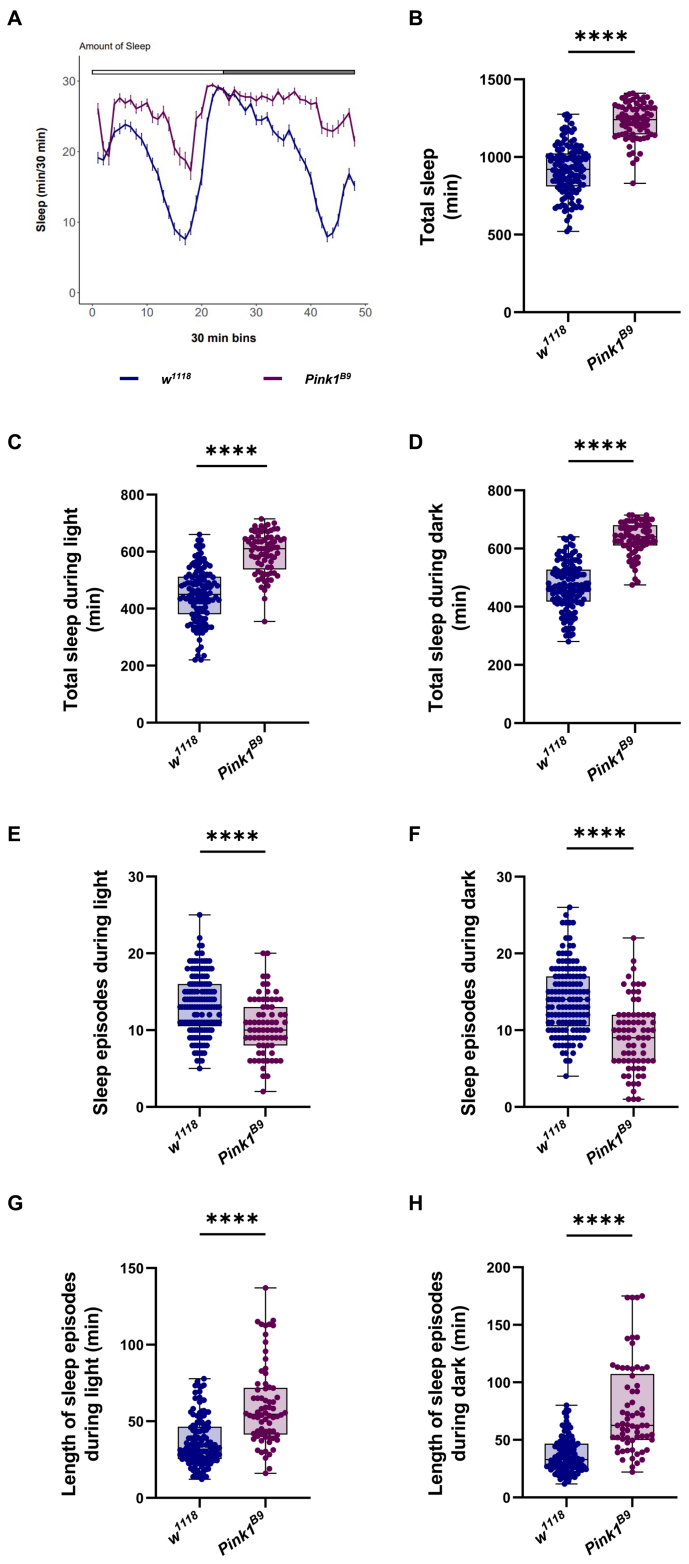
Drosophila *Pink1* mutant (KO) flies display aberrant sleep behavior. **(A)** Average daily sleep profile of *Pink1* KO flies compared to control from the second day of LD 12:12. **(B)** Total sleep amount (in minutes) during the second day of LD 12:12 conditions. **(C)** Sleep amount (in minutes) in the day/light phase of LD 12:12 conditions. **(D)** Sleep amount (in minutes) in the night/dark phase of LD 12:12 conditions. **(E)** Number of sleep episodes in the light/day phase of LD 12:12 conditions. **(F)** Number of sleep episodes in the dark/night phase of LD 12:12 conditions. **(G)** Length of the episodes of sleep (in minutes) in the light/day phase of LD 12:12 conditions. **(H)** Length of the episodes of sleep (in minutes) in the dark/night phase of LD 12:12 conditions. Sleep analysis was performed on rhythmic flies. *w^1118^ n* = 137; *Pink1^B9^ n* = 73.

Because the circadian clock controls sleep behavior, next we wanted to assess sleep–wake cycle in *Pink1* KO flies. To this aim, we monitored sleep behavior of flies of the indicated genotypes using the DAM monitoring system (Rosato and Kyriacou, 2006; Julienne et al., 2017). Sleep episodes in flies are defined as time in which flies do not change their position for at least 5 min (Schlichting et al., 2016). Recordings from the second day were analyzed to estimate sleep performance during the 24 h period (12 h of light and 12 h of darkness; Figure 3A). Wild type flies (*w^1118^*) exhibited characteristic sleep behavior: they seemed to be relatively active during the day and inactive at night, with a peak of inactivity during the day (“*siesta*”), 4 to 5 h after light went on (Figure 3A, blue line). On the contrary, *Pink1* mutants seemed to sleep a lot more, especially during the day (Figure 3A, magenta line).

Consistent with this result, we observed a significant increase in total sleep in *Pink1* mutant flies (Figure 3B), either during the light phase, and in the dark phase (Figures 3C,D, respectively). *Pink1^B9^* flies displayed fewer episodes of sleep (Figures 3E,F), and longer episodes of sleep in both light and dark phases (Figures 3G,H).

Conclusively, *Pink1^B9^* mutant flies display an obvious impairment in their circadian behavior. In LD conditions, they seem to be less active during the day, with disrupted morning and evening anticipation. In constant dark conditions, a smaller percentage of flies were rhythmic, and displayed a significantly longer circadian period. *Pink1^B9^* flies seem to sleep more compared to wild type. The quality of their sleep appeared to be significantly affected, in that in *Pink1* mutants, sleep is less fragmented compared to WT, with flies displaying fewer but longer episodes of sleep.

### 3.3 *Usp14* down-regulation in *Pink1* mutant (KO) flies rescues the circadian and sleep defects of these flies

**Figure 4:**
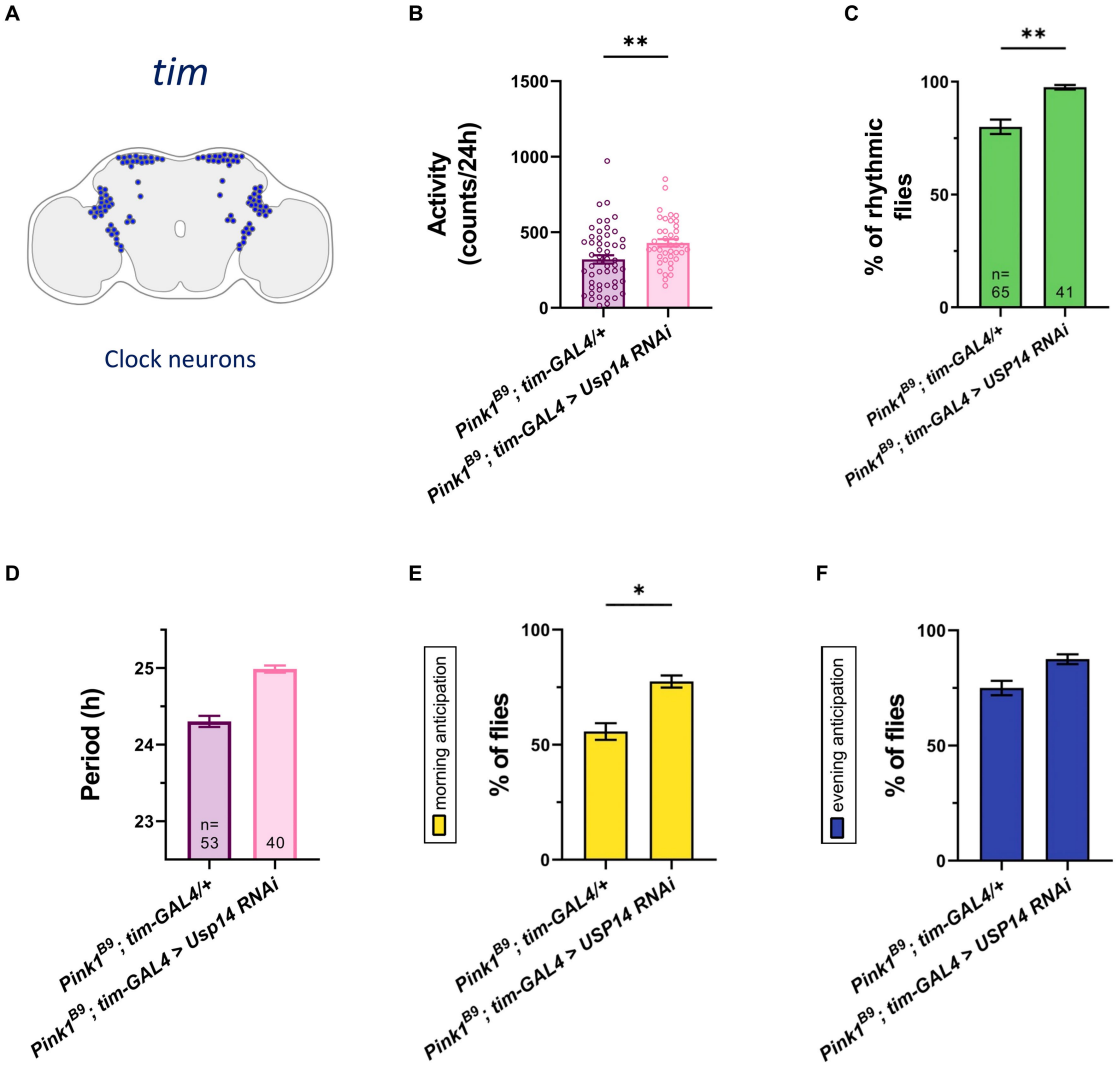
Tim-specific genetic down-regulation of *Usp14* in *Pink1* mutant flies rescues the circadian defects of *Pink1* KO flies. **(A)** Diagram of the clock neuron subset targeted by *Usp14 RNAi* using *tim-GAL4*. **(B)** Bar chart showing total activity during the second day of locomotor activity recording in LD. Charts shows total activity for each genotype as beam crosses per 24hrs. **(C)** Bar charts showing percentage of rhythmic flies. Number of total flies (rhythmic and arrhythmic) for each genotype is indicated. **(D)** Bar chart showing the period of rhythmic flies with clock neurons-specific inhibition of *Usp14*. **(E)** Bar charts indicating the percentage of flies that show morning anticipation (MA). **(F)** Bar charts indicating the percentage of flies that show evening anticipation (EA). Analysis was performed on rhythmic flies for **(B)**, **(E)** and **(F)**, and number of flies for each genotype is indicated in **(D)**.

*Pink1* mutant (KO) flies displayed reduced climbing and flight ability, shorter lifespan, and degeneration of the muscle of the thorax. The mutant genotype also develops mitochondrial dysfunction, and loss of DA neurons in the posterior protocerebrum lateral 1 (PPL1) cluster of the Drosophila brain. Pharmacological inhibition or genetic down-regulation of *Usp14* activity normalizes the abovementioned phenotypes, and lengthens *Pink1* KO lifespan to control conditions (Chakraborty et al., 2018). With that in mind, we wanted to assess whether *Usp14* down-regulation can also effectively recover the circadian phenotype associated to *Pink1* KO flies. We down-regulated *Usp14* in the tim expressing neurons (Figure 4A), as our data presented above indicate that *Usp14* inhibition had the most readable effect in this specific subset of circadian neurons (Figure 1). Flies of the desired genotypes were tested with the DAM apparatus for locomotor and circadian behavior, as described. We monitored total activity (Figure 4B), percentage of rhythmicity (Figure 4C), periodicity of the rhythmic flies, MA and EA (Figures 4D–F) of *Pink1* KO flies in which *Usp14* was down-regulated. We observed a rescue in the total activity of the *Pink1* KO flies (Figure 4B), and in all the circadian parameters that we tested (Figures 4C–E), except for the period (Figure 4D), which, as observed for wild type flies, continued to be lengthened. Down-regulation of *Usp14* in the whole animal with the *Actin* promoter (Supplementary Figure 2A) also seemed to be effective in preventing the development of the circadian phenotype of the *Pink1* KO flies, in that it rescued total activity, circadian rhythmicity, and morning anticipation (Supplementary Figures 1B–F).

**Figure 5:**
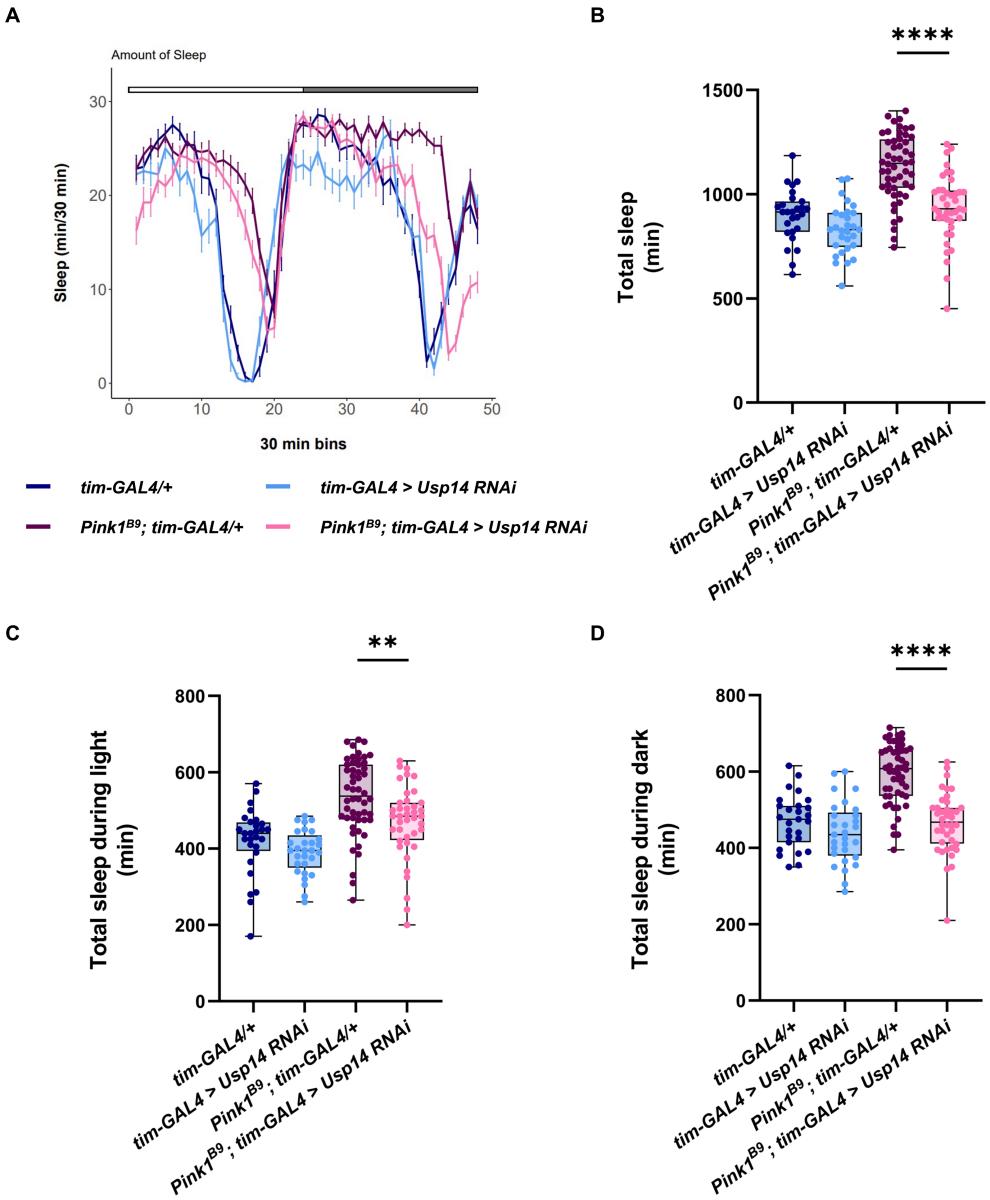
Tim-specific genetic down-regulation of *Usp14* in *Pink1* mutant flies rescues sleep defects of *Pink1* KO flies. **(A)** Average daily sleep profile of the indicated genotypes from the second day of LD 12:12. **(B)** Total sleep amount (in minutes) during the day of LD 12:12 conditions. **(C)** Sleep amount (in minutes) in the light/day phase of LD 12:12 conditions. **(D)** Sleep amount (in minutes) in the dark/night phase of LD 12:12 conditions. Sleep analysis was performed on rhythmic flies. *Tim-GAL4/+ n* = 26; *tim-GAL4 > USP14 RNAi n* = 29; *Pink1^B9^*; *tim-GAL4/+ n* = 53; *Pink1^B9^*; *tim-GAL4 > USP14 RNAi n* = 40.

Next, we wanted to investigate whether *Usp14* down-regulation also affects the sleep behavior of *Pink1* KO flies. Recordings from the second day were analyzed, and we estimated sleep performance during 12 h of light and 12 h of darkness of *Pink1* KO flies in which *Usp14* was down-regulated in tim + neurons. Down-regulation of *Usp14* completely rescued sleep performance of *Pink1* KO flies. In particular, they almost completely phenocopied wild type flies: they were active during the day, and rested during the night (Figure 5A). In perfect agreement with the sleep plot described in Figure 5A, quantification of total sleep showed that *Usp14* down-regulation in tim + neurons completely normalized sleep behavior of *Pink1* KO flies (Figures 5B–D).

In summary, down-regulation of *Usp14* in *Pink1^B9^* flies normalizes the circadian defects, and the sleep behavior of these flies.

## 4 Discussion

Parkinson’s disease (PD) is a multifactorial devastating degenerative disease of the central nervous system, which affects around 0.3% of the population (Raza et al., 2019). PD is characterized by intracellular accumulation of α-synuclein aggregates that form the Lewy bodies (LB) in the *substantia nigra* of the brain, and cause the selective loss of dopaminergic neurons with consequently depleted dopamine levels (Dauer and Przedborski, 2003; Mullin and Schapira, 2015). Motor dysfunctions such as bradykinesia, resting tremor, muscular rigidity, and postural instability are the main symptoms that characterize PD (Litvan et al., 2003).

Besides motor impairments, non-motor dysfunctions are commonly present in PD patients, and they include neuropsychiatric symptoms (depression, cognitive dysfunctions and dementia; Biundo et al., 2016), autonomic symptoms (bladder disorders, orthostatic hypotension, erectile impotence; Chaudhuri and Schapira, 2009), and disturbances in sleep–wake cycle (Iranzo et al., 2006). These non-motor symptoms (NMSs) have gained increasing relevance for their impact on the quality of life of PD patients, and their contribution to institutionalization at advanced disease. They are not only common in early PD, but they can also manifest before the onset of the classical motor symptoms. Circadian and sleep disturbances in particular can precede the onset of the cognitive decline and motor symptoms by many years, suggesting that disruption of circadian rhythm might not simply be a consequence of PD but a pathogenic component of the disease that can lead to or at least contribute to neurodegeneration (Videnovic and Golombek, 2013).

The mechanistic link between altered circadian rhythms and PD is poorly understood, but proposed underlying mechanisms suggest alterations in protein and organelle homeostasis (i.e., proteostasis; Leng et al., 2019). Proteostasis is gradually lost during physiological aging. In neurodegenerative diseases, like PD, loss of proteostasis is exacerbated, and depends on the pathological decay in the proteolytic activity of the ubiquitin-proteasome and the lysosome-autophagy system, the two major degradative systems of the cell (Bennett et al., 2005; Komatsu et al., 2006). Indeed, PD is characterized by accumulation of misfolded proteins and dysfunctional mitochondria, which can be at least in part ascribed to defective mechanisms of proteostasis (Cuervo et al., 2004). Thus, promoting the activity of the UPS or autophagy is likely to be beneficial in models of PD because it enhances the degradation of misfolded proteins and dysfunctional organelles, which can be sources of oxidative stress and cellular damage (McNaught et al., 2004). In this scenario, the “proteostatic” effect of Usp14 inhibition can be a valuable therapeutic approach, due to its unique ability to both enhance the UPS (Hanna et al., 2006; Lee et al., 2010, 2016), autophagy (Xu et al., 2016; Kim et al., 2018) and mitophagy (Chakraborty et al., 2018).

We previously reported that genetic down-regulation or pharmacological inhibition of Usp14 in *Pink1* and *Parkin* KO flies extended flies lifespan, and rescued the locomotor phenotype associated with these flies (Chakraborty et al., 2018), presumably by promoting proteostasis. In the current work we wanted to extend these findings, and address the potential beneficial effect of *Usp14* inhibition in the context of circadian dysfunction in *Pink1* KO flies modeling PD.

*Pink1* mutants develop very obvious circadian defects. In particular, Julienne H. and co-authors showed a decrease in total activity in *Pink1* KO flies, and a significant reduction in rhythmicity. Among those flies that remained rhythmic, authors did not observe a significant difference in the circadian period (Julienne et al., 2017). A parallel study reported a trend toward a decrease in total activity of *Pink1* KO flies, while a significant decrease in total activity was shown when *Pink1* was specifically down-regulated in *elav* expressing cells. In this study, *Pink1* KD flies displayed increased daytime sleep, whereas night-time sleep was not affected (Doktor et al., 2019). In support of these studies, we also observed defective circadian behavior in *Pink1^B9^* mutant (*Pink* KO) flies. In LD conditions, *Pink1* KO flies are less active during the day, with reduced ability to anticipate morning and evening clues. The percentage of rhythmic flies was significantly reduced in *Pink1^B9^* mutant flies. Among those flies that remained rhythmic, the circadian period was significantly lengthened. We also found that *Pink1^B9^* flies sleep significantly more during the night and during the day compared to wild type. The quality of their sleep appeared to be different compared to wild type flies. In particular, *Pink1* KO displayed fewer but longer episodes of sleep during the night and during the day (i.e., longer “*siesta*”).

Our results showed that down-regulation of *Usp14* completely rescued the circadian phenotype of *Pink1* KO mutants, and the sleep defects of these flies, while *Usp14* down-regulation alone in wild type background did not seem to affect the circadian clock, with some mild effect that was readable only in clock-relevant neurons.

The molecular mechanism by which Usp14 ameliorates PD circadian symptoms in flies is unknown. It may be direct, by altering protein stability of core components of the circadian clock, or indirect. It is known that in mammals Usp14 affects the circadian period by slowing down the degradation of clock protein Period (PER; D'Alessandro et al., 2017). However, the mild effect of *Usp14* down-regulation on the circadian phenotype of wild type flies suggests that protein level of core components of the clock is not significantly affected by Usp14 deubiquitinating activity, although this circumstance cannot be excluded. Another possibility is that Usp14 rescues circadian defects of *Pink1* KO flies by an indirect mechanism, for example by promoting mechanisms of mitochondrial quality control that relies on mitochondrial dynamics. It is known that promotion of mitochondrial fission rescues the aberrant phenotype and circadian defects associated with mice lacking a functional clock (Jacobi et al., 2015), and recovers disrupted circadian oscillation of oxidative phosphorylation and mitochondrial dysfunction of cells with specific ablation of clock gene *BMAL1* (Jacobi et al., 2015; Li et al., 2020). Intriguingly, cells lacking mitochondrial fission do not display circadian oscillation of core component of the circadian clock (Schmitt et al., 2018), suggesting that dysregulation in mitochondrial dynamics likely feedbacks to the circadian clockwork. Because *Usp14* down-regulation promotes mitochondrial fission and mitophagy (Chakraborty et al., 2018), it is tempting to speculate that the normalization of the circadian defects may arise from the pro-fission effect of *Usp14* down-regulation, which is key to mitochondrial quality control.

Clinical diagnosis of PD is mainly based on the presence of motor symptoms, which usually appear when more than 50% of dopaminergic neurons have already been lost. Since the diagnosis occurs too late for neuroprotective treatments to work, it is important to find a way to diagnose the disease before the appearance of motor symptoms, and implement a therapy at the premotor phase to prevent or delay the development and progression of disease (Kalia and Lang, 2015). In this respect, sleep disturbances, as well as non-motor symptoms in general, may offer a pre-symptomatic window for the treatment of this disease, allowing earlier intervention (Julienne et al., 2017). In this premotor phase, the pathogenic process of PD is presumed to be underway, involving regions of the peripheral and central nervous system that are distinct from the dopaminergic neurons of the *substantia nigra pars compacta* (SNpc). At present we do not know whether improving sleep and circadian behavior early on in PD patients may delay or even prevent the progression of the diseases. Circadian and locomotor dysfunction appears early in *Pink1* KO flies, and they are tested when flies are relatively young (3 to 6 days). In this respect, the fly model does not fully recapitulate the progression of the disease in human patients, where, particularly in the sporadic forms, there is a time gap between the appearance of the locomotor and the circadian-related symptoms. Thus, we cannot address this relevant hypothesis in flies, but it could be in principle addressed in mice. There are at least two murine models, the 6-hydroxydopamine (6-OHDA) model (Simola et al., 2007) and the MitoPark mouse (Galter et al., 2010), where circadian rhythm and sleep behavior have been fully characterized. Both models show a reduction of locomotor activity, accompanied by a severe disruption of circadian rhythm in constant darkness, which are indicative of parallel sleep dysfunctions (Fifel and Cooper, 2014; Masini et al., 2017).

Importantly, highly specific and potent small-molecule inhibitors of Usp14 are available, which are orally bioavailable, can cross the blood–brain barrier (BBB), and are well-tolerated in murine models. Usp14 inhibitors (IU1 and IU1-47) were originally developed some years ago (Lee et al., 2010; Boselli et al., 2017), and continue to be optimized from the original backbone. Given their high specificity (Wang et al., 2018), the development of therapeutic approaches targeting Usp14 seems to be a feasible therapeutic avenue.

In summary, in this work we strengthened Drosophila as animal model for investigating sleep and circadian symptoms of PD, and extended the evidences on the protective effect of *Usp14* down-regulation in the context of circadian and sleep disturbances.

## Data availability statement

The original contributions presented in the study are included in the article/Supplementary material, further inquiries can be directed to the corresponding author.

## Ethics statement

The manuscript presents research on animals that do not require ethical approval for their study.

## Author contributions

MF: Data curation, Writing – review & editing, Investigation, Methodology, Visualization. SM: Investigation, Methodology, Writing – review & editing, Formal analysis, Supervision. GB: Formal analysis, Investigation, Writing – review & editing. MZ: Formal analysis, Writing – review & editing. GM: Formal analysis, Writing – review & editing, Conceptualization, Data curation, Methodology, Supervision. EZ: Conceptualization, Data curation, Formal analysis, Supervision, Writing – review & editing, Funding acquisition, Resources, Writing – original draft.

## Supplementary material

SUPPLEMENTARY FIGURE S1 Actin-driven genetic down-regulation of Usp14 **(A)** Diagram of tissues targeted for down-regulation of Usp14 using Act-GAL4. **(B)** Total RNA was extracted from flies of the indicated genotypes, and retrotranscribed into cDNA. Specific Usp14 and endogenous control oligonucleotides primers were used to perform quantitative RT-PCR. Bar graph indicates Usp14 mRNA levels relatively to endogenous control in Usp14 RNAi induced flies, as indicated.

SUPPLEMENTARY FIGURE S2 Actin-driven genetic down-regulation of Usp14 in Pink1 mutant flies partially rescues the circadian phenotype of these flies. **(A)** Diagram of tissues targeted for down-regulation of Usp14 using Act-GAL4. **(B)** Bar chart showing total activity during the second day of locomotor activity recording in LD. Chart shows total activity for each genotype as beam crosses, per 24hrs. **(C)** Bar chart showing percentage of rhythmic flies. Number of total flies (rhythmic and arrhythmic) for each genotype is indicated. **(D)** Bar chart showing the period of rhythmic flies upon down-regulation of Usp14. **(E)** Bar chart indicating the percentage of flies that shows morning anticipation (MA). **(F)** Bar chart indicating the percentage of flies that shows evening anticipation (EA). Analysis was performed on rhythmic flies for **(B)**, **(E)** and **(F)**, and number of flies for each genotype is indicated in **(D)**.

## References

[ref1] Bennett E. J. Bence N. F. Jayakumar R. Kopito R. R. (2005). Global impairment of the ubiquitin-proteasome system by nuclear or cytoplasmic protein aggregates precedes inclusion body formation. Mol. Cell 17, 351–365. doi: 10.1016/j.molcel.2004.12.021 15694337

[ref2] Biundo R. Weis L. Antonini A. (2016). Cognitive decline in Parkinson's disease: the complex picture. NPJ Parkinsons Dis 2:16018. doi: 10.1038/npjparkd.2016.18, PMID: 28725699 PMC5516581

[ref3] Blesa J. Trigo-Damas I. Quiroga-Varela A. Jackson-Lewis V. R. (2015). Oxidative stress and Parkinson's disease. Front. Neuroanat. 9:91. doi: 10.3389/fnana.2015.00091 26217195 PMC4495335

[ref4] Boselli M. Lee B. H. Robert J. Prado M. A. Min S. W. Cheng C.. (2017). An inhibitor of the proteasomal deubiquitinating enzyme USP14 induces tau elimination in cultured neurons. J. Biol. Chem. 292, 19209–19225. doi: 10.1074/jbc.M117.815126, PMID: 28972160 PMC5702663

[ref5] Chakraborty J. von Stockum S. Marchesan E. Caicci F. Ferrari V. Rakovic A.. (2018). USP14 inhibition corrects an in vivo model of impaired mitophagy. EMBO Mol. Med. 10:e9014. doi: 10.15252/emmm.201809014, PMID: 30249595 PMC6220287

[ref6] Chaudhuri K. R. Schapira A. H. (2009). Non-motor symptoms of Parkinson's disease: dopaminergic pathophysiology and treatment. Lancet Neurol. 8, 464–474. doi: 10.1016/S1474-4422(09)70068-7 19375664

[ref7] Cuervo A. M. Stefanis L. Fredenburg R. Lansbury P. T. Sulzer D. (2004). Impaired degradation of mutant alpha-synuclein by chaperone-mediated autophagy. Science 305, 1292–1295. doi: 10.1126/science.1101738, PMID: 15333840

[ref8] D'Alessandro M. Beesley S. Kim J. K. Jones Z. Chen R. Wi J.. (2017). Stability of wake-sleep cycles requires robust degradation of the PERIOD protein. Curr. Biol. 27, 3454–3467.e8. doi: 10.1016/j.cub.2017.10.014, PMID: 29103939 PMC5698108

[ref9] Dauer W. Przedborski S. (2003). Parkinson's disease: mechanisms and models. Neuron 39, 889–909. doi: 10.1016/S0896-6273(03)00568-3 12971891

[ref10] Doktor B. Damulewicz M. Pyza E. (2019). Effects of MUL1 and PARKIN on the circadian clock, brain and behaviour in Drosophila Parkinson's disease models. BMC Neurosci. 20:24. doi: 10.1186/s12868-019-0506-8, PMID: 31138137 PMC6540415

[ref11] Fifel K. Cooper H. M. (2014). Loss of dopamine disrupts circadian rhythms in a mouse model of Parkinson's disease. Neurobiol. Dis. 71, 359–369. doi: 10.1016/j.nbd.2014.08.024, PMID: 25171792

[ref12] Galter D. Pernold K. Yoshitake T. Lindqvist E. Hoffer B. Kehr J.. (2010). MitoPark mice mirror the slow progression of key symptoms and L-DOPA response in Parkinson's disease. Genes Brain Behav. 9, 173–181. doi: 10.1111/j.1601-183X.2009.00542.x, PMID: 20002202 PMC4154513

[ref13] Giorgi C. Marchi S. Pinton P. (2018). The machineries, regulation and cellular functions of mitochondrial calcium. Nat. Rev. Mol. Cell Biol. 19, 713–730. doi: 10.1038/s41580-018-0052-8 30143745

[ref14] Greene J. C. Whitworth A. J. Kuo I. Andrews L. A. Feany M. B. Pallanck L. J. (2003). Mitochondrial pathology and apoptotic muscle degeneration in Drosophila parkin mutants. Proc. Natl. Acad. Sci. USA 100, 4078–4083. doi: 10.1073/pnas.0737556100, PMID: 12642658 PMC153051

[ref15] Hales K. G. Korey C. A. Larracuente A. M. Roberts D. M. (2015). Genetics on the Fly: a primer on the Drosophila model system. Genetics 201, 815–842. doi: 10.1534/genetics.115.183392, PMID: 26564900 PMC4649653

[ref16] Hanna J. Hathaway N. A. Tone Y. Crosas B. Elsasser S. Kirkpatrick D. S.. (2006). Deubiquitinating enzyme Ubp6 functions noncatalytically to delay proteasomal degradation. Cell 127, 99–111. doi: 10.1016/j.cell.2006.07.038, PMID: 17018280

[ref17] Hardin P. E. (2011). Molecular genetic analysis of circadian timekeeping in Drosophila. Adv. Genet. 74, 141–173. doi: 10.1016/B978-0-12-387690-4.00005-2, PMID: 21924977 PMC4108082

[ref18] Hernandez D. G. Reed X. Singleton A. B. (2016). Genetics in Parkinson disease: Mendelian versus non-Mendelian inheritance. J. Neurochem. 139, 59–74. doi: 10.1111/jnc.13593, PMID: 27090875 PMC5155439

[ref19] Hunt J. Coulson E. J. Rajnarayanan R. Oster H. Videnovic A. Rawashdeh O. (2022). Sleep and circadian rhythms in Parkinson's disease and preclinical models. Mol. Neurodegener. 17:2. doi: 10.1186/s13024-021-00504-w, PMID: 35000606 PMC8744293

[ref20] Iranzo A. Molinuevo J. L. Santamaria J. Serradell M. Marti M. J. Valldeoriola F.. (2006). Rapid-eye-movement sleep behaviour disorder as an early marker for a neurodegenerative disorder: a descriptive study. Lancet Neurol. 5, 572–577. doi: 10.1016/S1474-4422(06)70476-8, PMID: 16781987

[ref21] Jacobi D. Liu S. Burkewitz K. Kory N. Knudsen N. H. Alexander R. K.. (2015). Hepatic Bmal1 regulates rhythmic mitochondrial dynamics and promotes metabolic fitness. Cell Metab. 22, 709–720. doi: 10.1016/j.cmet.2015.08.006, PMID: 26365180 PMC4598294

[ref22] Julienne H. Buhl E. Leslie D. S. Hodge J. J. L. (2017). Drosophila PINK1 and parkin loss-of-function mutants display a range of non-motor Parkinson's disease phenotypes. Neurobiol. Dis. 104, 15–23. doi: 10.1016/j.nbd.2017.04.014, PMID: 28435104 PMC5469398

[ref23] Kalia L. V. Lang A. E. (2015). Parkinson's disease. Lancet 386, 896–912. doi: 10.1016/S0140-6736(14)61393-3 25904081

[ref24] Kang J. E. Lim M. M. Bateman R. J. Lee J. J. Smyth L. P. Cirrito J. R.. (2009). Amyloid-beta dynamics are regulated by orexin and the sleep-wake cycle. Science 326, 1005–1007. doi: 10.1126/science.1180962, PMID: 19779148 PMC2789838

[ref25] Kim E. Park S. Lee J. H. Mun J. Y. Choi W. H. Yun Y.. (2018). Dual function of USP14 Deubiquitinase in cellular proteasomal activity and Autophagic flux. Cell Rep. 24, 732–743. doi: 10.1016/j.celrep.2018.06.058 30021169

[ref26] Komatsu M. Waguri S. Chiba T. Murata S. Iwata J. Tanida I.. (2006). Loss of autophagy in the central nervous system causes neurodegeneration in mice. Nature 441, 880–884. doi: 10.1038/nature04723 16625205

[ref27] Lee B. H. Lee M. J. Park S. Oh D. C. Elsasser S. Chen P. C.. (2010). Enhancement of proteasome activity by a small-molecule inhibitor of USP14. Nature 467, 179–184. doi: 10.1038/nature09299, PMID: 20829789 PMC2939003

[ref28] Lee B. H. Lu Y. Prado M. A. Shi Y. Tian G. Sun S.. (2016). USP14 deubiquitinates proteasome-bound substrates that are ubiquitinated at multiple sites. Nature 532, 398–401. doi: 10.1038/nature17433, PMID: 27074503 PMC4844788

[ref29] Leng Y. Musiek E. S. Hu K. Cappuccio F. P. Yaffe K. (2019). Association between circadian rhythms and neurodegenerative diseases. Lancet Neurol. 18, 307–318. doi: 10.1016/S1474-4422(18)30461-7, PMID: 30784558 PMC6426656

[ref30] Li E. Li X. Huang J. Xu C. Liang Q. Ren K.. (2020). BMAL1 regulates mitochondrial fission and mitophagy through mitochondrial protein BNIP3 and is critical in the development of dilated cardiomyopathy. Protein Cell 11, 661–679. doi: 10.1007/s13238-020-00713-x, PMID: 32277346 PMC7452999

[ref31] Litvan I. Bhatia K. P. Burn D. J. Goetz C. G. Lang A. E. McKeith I.. (2003). Movement disorders society scientific issues, movement disorders society scientific issues committee report: SIC task force appraisal of clinical diagnostic criteria for parkinsonian disorders. Mov. Disord. 18, 467–486. doi: 10.1002/mds.10459, PMID: 12722160

[ref32] Ma D. Panda S. Lin J. D. (2011). Temporal orchestration of circadian autophagy rhythm by C/EBPbeta. EMBO J. 30, 4642–4651. doi: 10.1038/emboj.2011.322, PMID: 21897364 PMC3243590

[ref33] Masini D. Lopes-Aguiar C. Bonito-Oliva A. Papadia D. Andersson R. Fisahn A.. (2017). The histamine H3 receptor antagonist thioperamide rescues circadian rhythm and memory function in experimental parkinsonism. Transl. Psychiatry 7:e1088. doi: 10.1038/tp.2017.58, PMID: 28398338 PMC5416699

[ref34] Matheoud D. Sugiura A. Bellemare-Pelletier A. Laplante A. Rondeau C. Chemali M.. (2016). Parkinson's disease-related proteins PINK1 and Parkin repress mitochondrial antigen presentation. Cell 166, 314–327. doi: 10.1016/j.cell.2016.05.039, PMID: 27345367

[ref35] McNaught K. S. Perl D. P. Brownell A. L. Olanow C. W. (2004). Systemic exposure to proteasome inhibitors causes a progressive model of Parkinson's disease. Ann. Neurol. 56, 149–162. doi: 10.1002/ana.20186, PMID: 15236415

[ref36] Mullin S. Schapira A. H. (2015). Pathogenic mechanisms of neurodegeneration in Parkinson disease. Neurol. Clin. 33, 1–17. doi: 10.1016/j.ncl.2014.09.010 25432720

[ref37] Narendra D. P. Jin S. M. Tanaka A. Suen D. F. Gautier C. A. Shen J.. (2010). PINK1 is selectively stabilized on impaired mitochondria to activate Parkin. PLoS Biol. 8:e1000298. doi: 10.1371/journal.pbio.1000298, PMID: 20126261 PMC2811155

[ref38] Park J. Lee S. B. Lee S. Kim Y. Song S. Kim S.. (2006). Mitochondrial dysfunction in Drosophila PINK1 mutants is complemented by parkin. Nature 441, 1157–1161. doi: 10.1038/nature04788, PMID: 16672980

[ref39] Patke A. Young M. W. Axelrod S. (2020). Molecular mechanisms and physiological importance of circadian rhythms. Nat. Rev. Mol. Cell Biol. 21, 67–84. doi: 10.1038/s41580-019-0179-2 31768006

[ref40] Raza C. Anjum R. Shakeel N. U. A. (2019). Parkinson's disease: mechanisms, translational models and management strategies. Life Sci. 226, 77–90. doi: 10.1016/j.lfs.2019.03.057, PMID: 30980848

[ref41] Rosato E. Kyriacou C. P. (2006). Analysis of locomotor activity rhythms in Drosophila. Nat. Protoc. 1, 559–568. doi: 10.1038/nprot.2006.79 17406282

[ref42] Ryzhikov M. Ehlers A. Steinberg D. Xie W. Oberlander E. Brown S.. (2019). Diurnal rhythms spatially and temporally organize autophagy. Cell Rep. 26, 1880–1892.e6. doi: 10.1016/j.celrep.2019.01.072, PMID: 30759397 PMC6442472

[ref43] Schlichting M. Menegazzi P. Lelito K. R. Yao Z. Buhl E. Dalla Benetta E.. (2016). A neural network underlying circadian entrainment and photoperiodic adjustment of sleep and activity in Drosophila. J. Neurosci. 36, 9084–9096. doi: 10.1523/JNEUROSCI.0992-16.2016, PMID: 27581451 PMC5005721

[ref44] Schmitt K. Grimm A. Dallmann R. Oettinghaus B. Restelli L. M. Witzig M.. (2018). Circadian control of DRP1 activity regulates mitochondrial dynamics and bioenergetics. Cell Metab. 27, 657–666.e5. doi: 10.1016/j.cmet.2018.01.011, PMID: 29478834

[ref45] Seluzicki A. Flourakis M. Kula-Eversole E. Zhang L. Kilman V. Allada R. (2014). Dual PDF signaling pathways reset clocks via TIMELESS and acutely excite target neurons to control circadian behavior. PLoS Biol. 12:e1001810. doi: 10.1371/journal.pbio.1001810, PMID: 24643294 PMC3958333

[ref46] Shokri-Kojori E. Wang G. J. Wiers C. E. Demiral S. B. Guo M. Kim S. W.. (2018). beta-amyloid accumulation in the human brain after one night of sleep deprivation. Proc. Natl. Acad. Sci. USA 115, 4483–4488. doi: 10.1073/pnas.1721694115, PMID: 29632177 PMC5924922

[ref47] Simola N. Morelli M. Carta A. R. (2007). The 6-hydroxydopamine model of Parkinson's disease. Neurotox. Res. 11, 151–167. doi: 10.1007/BF03033565 17449457

[ref48] Sliter D. A. Martinez J. Hao L. Chen X. Sun N. Fischer T. D.. (2018). Parkin and PINK1 mitigate STING-induced inflammation. Nature 561, 258–262. doi: 10.1038/s41586-018-0448-9, PMID: 30135585 PMC7362342

[ref49] Summa K. C. Jiang P. Gonzalez-Rodriguez P. Huang X. Lin X. Vitaterna M. H.. (2024). Disrupted sleep-wake regulation in the MCI-Park mouse model of Parkinson's disease. NPJ Parkinsons Dis 10:54. doi: 10.1038/s41531-024-00670-w, PMID: 38467673 PMC10928107

[ref50] Varshavsky A. (2012). Augmented generation of protein fragments during wakefulness as the molecular cause of sleep: a hypothesis. Protein Sci. 21, 1634–1661. doi: 10.1002/pro.2148, PMID: 22930402 PMC3527701

[ref51] Videnovic A. Golombek D. (2013). Circadian and sleep disorders in Parkinson's disease. Exp. Neurol. 243, 45–56. doi: 10.1016/j.expneurol.2012.08.018, PMID: 22935723 PMC3666169

[ref52] Wang Y. Jiang Y. Ding S. Li J. Song N. Ren Y.. (2018). Small molecule inhibitors reveal allosteric regulation of USP14 via steric blockade. Cell Res. 28, 1186–1194. doi: 10.1038/s41422-018-0091-x, PMID: 30254335 PMC6274642

[ref53] Xie L. Kang H. Xu Q. Chen M. J. Liao Y. Thiyagarajan M.. (2013). Sleep drives metabolite clearance from the adult brain. Science 342, 373–377. doi: 10.1126/science.1241224, PMID: 24136970 PMC3880190

[ref54] Xie Y. Tang Q. Chen G. Xie M. Yu S. Zhao J.. (2019). New insights into the circadian rhythm and its related diseases. Front. Physiol. 10:682. doi: 10.3389/fphys.2019.00682, PMID: 31293431 PMC6603140

[ref55] Xu D. Shan B. Sun H. Xiao J. Zhu K. Xie X.. (2016). USP14 regulates autophagy by suppressing K63 ubiquitination of Beclin 1. Genes Dev. 30, 1718–1730. doi: 10.1101/gad.285122.116, PMID: 27542828 PMC5002977

