# Supplementary material for: Usp14 down-regulation corrects sleep and circadian dysfunction of a Drosophila model of Parkinson’s disease
Source: Front Neurosci. 2024 Aug 5;18:1410139. doi: 10.3389/fnins.2024.1410139 (PMC11330830; doi:10.3389/fnins.2024.1410139)
Supplement: Supplementary file 3 [file Data_Sheet_1.DOCX]

Supplementary Material

***Usp14* down-regulation corrects sleep and circadian dysfunction of a *Drosophila* model of Parkinson’s Disease**

**Mariavittoria Favaro, Sofia Mauri, Greta Bernardo, Mauro Zordan, Gabriella M. Mazzotta and Elena Ziviani^*^**

Department of Biology, University of Padova, Padova, Italy.

*** Correspondence:**Elena Ziviani
elena.ziviani@unipd.it

Keywords: Usp14, Pink1, Drosophila, Circadian Clock, Sleep, mitochondrial fission

# Supplementary Figures and Tables

**Supplementary Table 1.** Description of mutant lines

| *Drosophila melanogaster* line | Source | Identifier |
| --- | --- | --- |
| w^1118^ | BDSC | RRID:BDSC_6326 |
| w, Pink1^B9^/FM7-GFP | (Park et al., 2006) | FBal0193144 |
| P{KK102888}VIE-260B | VDRC | KK-110227 |
| Act5cGAL4/CyO-GFP | Whitworth Lab | N/A |
| P{w[+mW.hs]=GawB}elav[C155] | BDSC | RRID:BDSC_458 |
| P{w[+mC]=Pdf-GAL4.P2.4}X, y[1] w[*] | BDSC | RRID:BDSC_6899 |
| y[1] w[*]; P{w[+mC]=GAL4-tim.E}62 | BDSC | RRID:BDSC_7126 |

## Supplementary Figures legend

**Supplementary Figure 1 Actin-driven genetic down-regulation of Usp14**

(A) Diagram of tissues targeted for down-regulation of Usp14 using Act-GAL4. (B) Total RNA was extracted from flies of the indicated genotypes, and retrotranscribed into cDNA. Specific Usp14 and endogenous control oligonucleotides primers were used to perform quantitative RT-PCR. Bar graph indicates Usp14 mRNA levels relatively to endogenous control in Usp14 RNAi induced flies, as indicated.

**Supplementary Figure 2 Actin-driven genetic down-regulation of Usp14 in Pink1 mutant flies partially rescues the circadian phenotype of these flies.** (A) Diagram of tissues targeted for down-regulation of Usp14 using *Act-GAL4*. (B) Bar chart showing total activity during the second day of locomotor activity recording in LD. Chart shows total activity for each genotype as beam crosses, per 24hrs. (C) Bar chart showing percentage of rhythmic flies. Number of total flies (rhythmic and arrhythmic) for each genotype is indicated. (D) Bar chart showing the period of rhythmic flies upon down-regulation of Usp14. (E) Bar chart indicating the percentage of flies that shows morning anticipation (MA). (F) Bar chart indicating the percentage of flies that shows evening anticipation (EA). Analysis was performed on rhythmic flies for (B), (E) and (F), and number of flies for each genotype is indicated in (D).
